# Reducing violence for adolescents and their parents in three disadvantaged communities in North India: a pilot implementation study of the Parwarish (PLH-Teens) parenting programme

**DOI:** 10.1136/bmjopen-2025-113646

**Published:** 2026-06-25

**Authors:** Kaaren Mathias, Pooja Pillai, Prabhudutt Nayak, Pratibha Milton Singh, Miguel San Sebastian

**Affiliations:** 1 Faculty of Health, University of Canterbury, Christchurch, Canterbury, New Zealand; 2 Burans Community Mental Health Programme, Herbertpur Christian Hospital, Herbertpur, Uttarakhand, India; 3 Chhatarpur Christian Hospital, Emmanuel Hospital Association, New Delhi, Delhi, India; 4 Community health and development programme, Emmanuel Hospital Association, New Delhi, Delhi, India; 5 Department of Epidemiology and Global Health, Umeå Universitet, Umeå, Sweden

**Keywords:** India, Adolescents, Parents, MENTAL HEALTH, Family

## Abstract

**ABSTRACT:**

**Objectives:**

To evaluate the pilot implementation of Parwarish (Parenting for Lifelong Health–Teens) programme on reducing violence against adolescents and improving parenting practices, mental health, gender attitudes, resilience and financial coping among families in disadvantaged communities in North India.

**Design:**

Pilot implementation study using a pre-post study design.

**Setting:**

Community-based implementation in three disadvantaged settings in North India, including urban informal settlements, rural agricultural areas and remote tribal communities.

**Participants:**

A total of 239 adolescents (aged 12–18 years) and 478 parents from 239 families were recruited using purposive and snowball sampling; all participants completed baseline assessment, with follow-up conducted within 6 weeks after intervention completion.

**Interventions:**

A 14-week group-based parenting intervention delivered weekly in community settings, focusing on communication, problem-solving, non-violent discipline and financial management, facilitated by trained community members.

**Primary and secondary outcome measures:**

Primary outcomes were parent and youth-reported maltreatment. Secondary outcomes included parenting practices, adolescent behaviour, mental health, resilience, gender-equitable attitudes and financial coping.

**Results:**

Significant improvements were observed across all outcomes. Among adolescents, positive parenting increased (β=13.7; 95% CI 12.43 to 14.98), youth behaviour improved (β=9.12; 95% CI 8.11 to 10.13), resilience increased (β=9.53; 95% CI 8.19 to 10.86) and harsh discipline decreased (β=−8.69; 95% CI −10.45 to −6.94). Among parents, positive parenting improved (β=10.6; 95% CI 9.43 to 11.85), youth behaviour improved (β=11.11; 95% CI 10.20 to 12.02), harsh discipline decreased (β=−10.60; 95% CI −12.23 to −8.96) and financial coping improved (β=−4.97; 95% CI −5.98 to −3.97).

**Conclusions:**

This low-cost, community-delivered pilot implementation of the Parwarish intervention was associated with improvements in parenting practices and in mental health and resilience among adolescents and parents. While causal inference is limited by the study design, the findings support further evaluation using controlled designs. It suggests potential for scale-up in similar low-resource settings.

**Trial registration number:**

ACTRN12622000858796.

**STRENGTHS AND LIMITATIONS OF THIS STUDY**

- This real-world, community-delivered intervention was implemented in low-income settings, supporting its feasibility and potential scalability.
- The study was conducted with a modest budget, enhancing its relevance for implementation in resource-constrained contexts.
- The absence of a control group limits causal inference regarding the observed changes.
- Clustering by site and the lack of adjustment for key design features (eg, baseline values) may have affected the precision and validity of estimates.
- Reliance on self-reported outcomes introduces potential measurement bias, although triangulation and confidentiality procedures were used to mitigate this risk.

## Introduction

 Over 1 billion children and young people experience violence with the highest rates occurring in North America, Asia and Africa, where at least 50% of youth experienced past year violence and 11% of all young people (including children) experience a lifetime prevalence of sexual violence.^1 2^ Key factors associated with violence against young people and children have been identified as lower household socioeconomic status, only completing primary school education of mothers and adults in the household (for those experiencing emotional violence) and being a girl (for sexual violence). Violence against children and young people worsens their physical and mental health outcomes for the rest of their lives, violates human rights and limits school attainment in the short term and decreases functioning throughout the lifespan.^3^,^5^ Recognising these impacts, preventing violence against children is one of the most urgent global health priorities and has been included as the Sustainable Development Goal 16.2.^6^

While families and parents can be the source of violence, they also often provide a supportive and safe place in which young people can flourish. The following five core aspects of parenting that promote youth development have been identified as: (1) creating a safe, nurturing and engaging home environment, (2) creating a positive learning environment, (3) providing consistent boundaries and discipline, (4) having reasonable expectations of young people and parents and (5) having the capacity for self-care in the parenting role.^7^

There is recent and compelling evidence of the value of positive parenting programmes to reduce violence against young people and to protect them against poor developmental outcomes. These programmes can work by strengthening communication, executive function, self-regulation, sibling and peer relationships and academic attainment. Parenting programmes have also been shown to improve mental and physical health^8 9^ and to address multiple upstream health determinants, including poverty reduction through strengthened household financial management and literacy, gender equity, access to education and resilience within families and communities.^9^

In India, people under 25 make up over 40% of the population,^10^ yet millions of young people across all socioeconomic groups experience diverse forms of violence, including early marriage, domestic abuse, sexual violence, trafficking, online violence, child labour and bullying.^1^ The prevalence of violence against young people under 18 years (including psychological, sexual and physical violence) ranges from around 21% to 50%.^1 5 11 12^ Violence occurs at higher rates in communities with lower incomes, limited maternal education and poor living conditions, and these childhood adversities can lead to neglect and abuse and often ‘cluster’.^5^ Young people living in low-income informal urban communities (representing one in six Indian youth) are at particular risk, as these areas have higher rates of poverty, child labour and child maltreatment.^5^

Four main pieces of legislation in India have been designed to protect children and young people: The Juvenile Justice (Care and Protection) Act (2000, amended in 2015); The Prohibition of Child Marriage Act (2006); The Protection of Children from Sexual Offences Act (2012); and The Child Labour (Prohibition and Regulation) Act (1986, amended in 2016). In 2019, the Protection of Children from Sexual Offences Bill was amended, stipulating stricter punishment for sexual crimes against children and young people. Yet while the policy and legal frameworks are comprehensive, there are significant challenges in implementing and regulating these laws due to sparse resources and support services.

The Parenting for Lifelong Health (PLH) programme is a group intervention that was developed in South Africa to reduce violence towards children in families. The development, implementation and adaptation of PLH for specific subgroups has been well documented,^813^,^17^ and a pragmatic randomised cluster-controlled study demonstrated the programme can significantly reduce physical and emotional abuse of young people as well as improve positive parenting and parental mental health.^18^ It has since been adapted and implemented in settings such as Tanzania, Philippines and China and has continued to show reduced rates of violence towards young people in families.^13 19 20^

Yet despite evidence of the value of parenting programmes and of widespread violence experiences for young people, a recent scoping review of positive parenting programmes among young people in low- and middle-income countries (LMIC) found that not one study had been implemented in India.^21^

The aim of this study was to assess the impact of the Parwarish programme on (a) reducing violence by parents in families; (b) improving mental health, gender attitudes and financial coping among parents; and (c) improving mental health, gender attitudes and resilience among young people in northern India.

## Methods

### Setting

A collaboration between UNICEF India, the Emmanuel Hospital Association (EHA; a non-profit organisation working across North and Northeast India) and Umeå University (Sweden) with funding from the Swedish Research Council led to the adaptation and pilot implementation of this intervention in North India in 2019. The adaptation of PLH-Teens was coproduced with women from a low-income informal urban community in Dehradun, North India, and described in the process evaluation.^22^

Recognising that randomised controlled trials (RCTs) are expensive, often not feasible and can fail to replicate real-life settings due to rigid processes required across implementation, we decided to conduct a pre-post evaluation of this pilot implementation among non-randomised participants.^23^ We carried out this study between April and October 2019 in three settings: (1) low-income informal urban settlements in the mega-city Agra in Uttar Pradesh; (2) a densely populated rural agricultural area in Sonbhadra district, Uttar Pradesh; and (3) small hamlets in the sparsely populated tribal region of Khunti, Jharkhand. Locations were selected based on previous community health and development programmes of at least 5 years duration as part of the community health and development programme of the EHA.

### Participant recruitment and group formation

Participants were recruited by facilitators through purposive sampling, by directly inviting known families with an adolescent living at home and subsequent snowball sampling. Inclusion criteria were residence in the target areas; having at least one adolescent aged 12–18 years; having one or both parents acting as primary caregivers (residing in the same household for at least four nights a week); and willingness to participate in the 2-hour weekly group sessions. We excluded families planning to migrate out of the target location during the intervention period.

**Figure 1:**
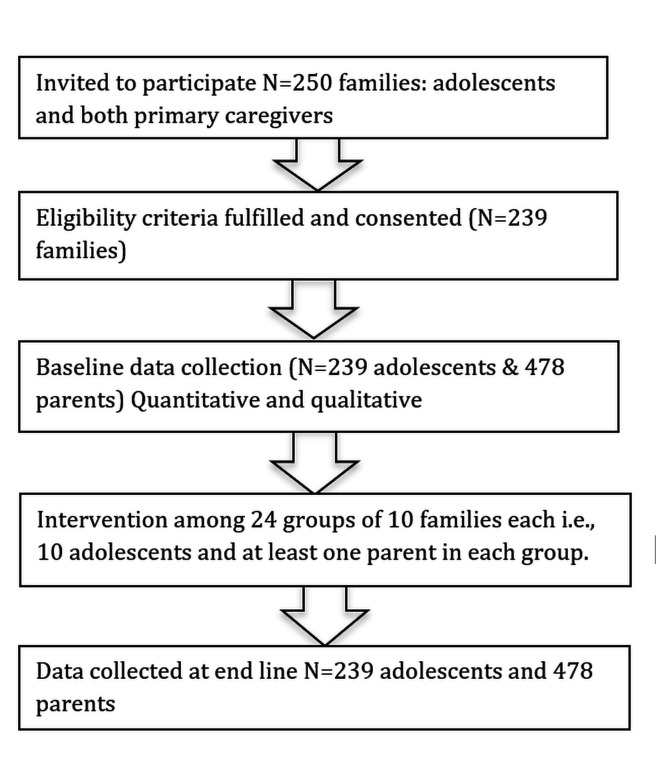
Overview of Parwarish intervention recruitment.

Both parents were recruited and in families with more than one adolescent, families chose which young person would participate. Parents gave consent, while young people gave assent for participation. Sample size was not calculated formally but pragmatically set at n=250 families to be invited. Of a total of 250 families approached, 239 families (n=239 adolescents and 478 parents) consented to participate. Groups were formed based on household proximity with 10 families per group, yielding a total of 24 groups. Figure 1 provides an overview of the recruitment and study participation.

### The intervention

Groups met weekly over a 14-week period at a mutually agreed time and location in the community, typically the courtyard of a host family. Facilitators followed an approach which included role-plays, games, emotional check-ins and home rehearsal activities to build parenting skills and training principles. Table 1 summarises the 14 modules of the Parwarish programme and their core content.

**Table 1:** Overview of Parwarish intervention content^22^

Module No	Core content	Focus for skill development in home activities
1–3	Building positive relationships—spending quality time with your family members, praising and giving positive feedback.	Spending time. Asking open-ended questions. Giving praise.
4	Identifying, naming and talking about emotions.	Naming emotions and where they are experienced.
5, 6	Managing anger and solving problems.	
7, 11	Making a budget and ways to save money and manage family finances.	Making a family budget.
8, 9, 10	Making family rules and solving problems without conflict.	Identifying problems and desired behaviour, practice alternatives to violent discipline, family rules.
12–14	Staying safe in the community, plans for managing crisis and developing circles of support.	Identifying safe people and places, processes for seeking help in crisis and identifying support.

### Implementation

The study was implemented between June and October 2019. All facilitators and coaches participated in a 7-day training on the programme’s broad content, delivered in Delhi by the PLH-Teens group from South Africa. This was followed by three further days of training for the coaches. This training addressed both content of the modules (working through each of the modules during these days) and covering skills in interactive facilitation and group leadership skills. There was 100% attendance by facilitators and coaches (n=25) as well as implementers. The implementation team comprised two programme coordinators (PMS and PN) and three coaches who were also project leaders at each site. This group met fortnightly to discuss implementation, address any concerns and codevelop implementation processes. Coaches selected group facilitators with these inclusion criteria: effective communication skills, fluency in the local language, an equal mix of men and women (over half of group facilitators working together were married couples), having completed at least class 10 at high school and resident in the target community. A total of 19 group facilitators were identified and each led one or two groups. Coaches visited facilitators and their group sessions at least once a fortnight to conduct monitoring and fidelity assessments. In addition, they provided weekly support to facilitators, including discussions of implementation challenges and training on intervention content throughout the trial. Further details on implementation, fidelity and processes are available in the published process evaluation paper.^22^

### Data collection

Tools were selected by the research and implementation teams based on validation in India, relevance to expected outcomes and tools used in other PLH-Teens evaluations.^15 18 24^ Coaches and data collectors (different from the facilitators) were trained in recruitment, taking consent and question interpretation by PP. Coaches trained the data collectors to use the psychometric scales in their specific context with support from PP and KM. Data were collected at baseline (pre-implementation) and post-endline (within 6 weeks of intervention completion). To accommodate limited literacy among participants, data collectors filled forms beside the parents and young people by reading the questions aloud.

### Psychometric tools

Table 2 summarises the standardised questionnaires used and the corresponding outcomes measured. The primary outcome was youth and parent reports of maltreatment (violence or abuse), measured using the ISPCAN Child Abuse Screening Tool (ICAST) and the Alabama Parenting Questionnaire (APQ). The ICAST^25^ has been successfully used in LMICs to measure violence or abusive disciplinary actions against children and young people (maximum score=80).^18^

**Table 2:** Summary of standardised questionnaires used for data collection*

Measure used	Outcome measured	Adolescent total question items	Parent total question items
**ISPCAN Child Abuse Screening Tool (child maltreatment)**	Discipline methods survey	15	15
**Alabama Parenting Questionnaire (parental involvement and supervision)**	Involved parenting and parental monitoring measures	20	20
**Strengths and Difficulties Questionnaire (child behaviour)**	Child behaviour measure	25	25
Patient Health Questionnaire-9 (depression)	Stress/depression	9	9
Family Financial Coping Scale	Economic strengthening	–	4
Gender Equity Measure (gender-equal attitudes)	Gender-equal attitudes	8	8
Child Youth Resilience Measure (resilience)	Resilience	12	–

* The primary outcomes are marked in bold.

ISPCAN, International Society for the Prevention of Child Abuse and Neglect.

Parent reports of positive parental involvement (maximum total score=40) and parental supervision (maximum total score=40) were assessed using subscales from the APQ (10 items each).^26^ Respondents reported on the frequency of parenting practices in the past month (eg, ‘You talk to your child about his/her friends’) using a 5-item Likert scale.

Youth behaviour was assessed using parent and youth reports on the *Strengths and Difficulties Questionnaire*^27^ (25 items, maximum total score=50), which has been validated in South Asia and used successfully as a self-report instrument in North India.^28^ It measures five behavioural and emotional subscales: emotional symptoms, conduct problems, hyperactivity, peer problems and prosocial. Internalising and externalising behaviour scores can be calculated by summing the emotional symptoms and peer problems for internalising and conduct problems and hyperactivity subscales for externalising.

The *Patient Health Questionnaire-9* (*PHQ-9*) (maximum total score=27) is a depression screening tool^29^ validated for use in India and among adolescents.^30^

The *Family Financial Coping Scale*^31^ uses six items to assess financial self-efficacy household economic/financial hardship using factors such as availability of money for basic necessities and status of loans taken in the previous weeks. It was validated in North India subsequent to our study.^32^

The *Gender Equitable Measure Scale* (maximum total score=32) measures attitudes towards gender equality and includes areas such as sexual and reproductive health and domestic violence^33^ and has been validated in India.^34^

The *Child Youth Resilience Measure* (maximum total score=60) is a tool used to assess the individual, social and emotional assets and resources available in and around children and young people and has been validated in India.^35 36^

*Sociodemographic data* collected included ownership of a ‘ration card’ (subsidised grains), below poverty line status (a Government of India classification), place of residence, caste, religion, marital status, housing quality, highest education attained, literacy and indebtedness, adapted from the Indian National and Family Health Surveys.^37^

While we could use an existing validated translation in Hindi of PHQ-9, other scales were translated into Hindi and back-translated for meaning by coaches and peer reviewed at all three locations to ensure accuracy.

### Data analysis

Cronbach’s alpha was first applied to examine the reliability (internal consistency) of the scales (online supplemental file 1). Most of the scales showed good to high reliability but were relatively poorer for parental supervision (both parents and young people) and for gender-equal attitudes (for young people).

Frequencies and percentages for the sociodemographic characteristics of the participants at baseline and mean and SD for each scale for both the preintervention and postintervention periods were calculated. Linear regression models were used to estimate the mean change in outcome scores between preintervention and postintervention measurements, with time (baseline vs endline) as the independent variable and outcome scores as dependent variables; baseline values were not included as covariates because they were time invariant. Analysis was conducted with SPSS V.26.

### Patient and public involvement

Community members in all three of the participating locations requested the implementing organisation (EHA) to implement a parenting project. Community members in urban Dehradun (a group of women resident in a low-income urban setting) collaborated with team leaders from the EHA to adapt the South African intervention PLH-Teens for an Indian setting, Parwarish. Programme intervention facilitators were also resident in the three target communities. Findings from this project were shared in dissemination meetings with all three participating communities.

### Positionality

The research team comprised PP, KM, PMS and PN (employed by host non-profit, EHA at this time) as well as MSS (a scholar based in Sweden supporting the India-based team through a research capacity building grant). Research design was developed by PP, KM and MSS with Isabel Goicolea, Umeå Universitet.

### Ethics approval and trial registration

We completed the clinical trial registration in June 2022 with the Australian New Zealand Clinical Trials Registry (ACTRN12622000858796).

## Findings

We present the sociodemographic profile of participants, fidelity (attendance) and changes in psychometric measures reported by young people and parents.

### Characteristics of the participants

The sociodemographic profile of participants is presented in table 3. The largest portion of families came from Agra (54.0%), and there were more boys (55.3%) than girls (44.7%) participating. The average age of adolescents and their parents was 13.4 and 39.1, respectively. Almost all families belonged to the more disadvantaged Scheduled Caste, Scheduled Tribe and ‘Other backward tribe’ groups. Other markers of disadvantage included over half of parents being illiterate and more than half of families living in houses made of temporary materials. A total of 45% of participating families were identified as below the poverty line (with an officially designated ID).

**Table 3:** Characteristics of participants at baseline

	Adolescent n (%)	Parents n (%)
Intervention site, state		
Agra, Uttar Pradesh (urban)	128 (54.0)	–
Khunti, Jharkhand (rural tribal)	49 (20.7)	–
Robertsganj, Uttar Pradesh (rural)	60 (25.3)	–
Gender of young people		
Girls	106 (44.7)	–
Boys	131 (55.3)	–
Age: mean (SD)	13.4 (2.5)	39.1 (7.0)
Caste		
SC/ST/ OBC	229 (96.6)	–
General	8 (3.4)	–
Religion		
Hindu	171 (72.2)	–
Other (Muslim, Christian and traditional)	66 (27.9)	–
Housing structure*		
Temporary materials (kaccha), for example, tarpaulin or bamboo roof and dirt floor	123 (51.9)	–
Semipermanent (semi-pucca), for example, tin roof and dirt floor	47 (19.9)	–
Permanent materials (pucca), for example, tin roof and concrete floor	64 (27.0)	–
Not recorded	3 (1.3)	–
Reading		
Illiterate	48 (20.3)	133 (56.1)
Literate	189 (79.8)	104 (43.9)
Card for below poverty line status (family)		108 (45.6)
No		129 (54.4)
Yes		108 (45.6)

* Materials used for housing construction which is a measure of socio-economic status in urban India.

OBC, Other Backward Classes; SC, Scheduled Castes; ST, Scheduled Tribes.

### Attendance

Mothers and young people participated actively and attended a mean of at least 11.0 out of 14 sessions. The mean number of sessions attended by fathers was poorer (5.8 of 14 sessions).

### Outcomes of psychometric measures

The mean scores at the two time points and the estimates of the crude and adjusted regression models are provided in table 4 for adolescents and table 5 for caregivers.

**Table 4:** Young persons’ outcomes at preintervention and postintervention and their differences (β coefficients) with 95% CIs estimated using linear regression models (n=239)

Outcome*	Pretest (T1)†	Post-test (T2)†	Estimated change β (95% CI)
Positive and involved parenting (40)	18.5 (8.6)	32.2 (5.2)	13.7 (12.43 to 14.98)
Parental monitoring (40)	23.8 (4.9)	29.1 (4.9)	5.25 (4.36 to 6.14)
Total behaviour (50)	33.2 (5.9)	42.3 (5.2)	9.12 (8.11 to 10.13)
Discipline caregiver (80)	12.6 (12.5)	3.9 (5.7)	−8.69 (−10.45 to −6.94)
Discipline method	13.3 (4.4)	14.8 (6.3)	1.52 (0.54 to 2.50)
Depression	5.93 (5.3)	2.3 (2.3)	−3.65 (−4.43 to −2.88)
Gender views and attitudes (32)	26.0 (6.0)	28.1 (3.4)	2.04 (1.16 to 2.02)
Adolescent resilience (60)	43.7 (9.2)	53.2 (5.1)	9.53 (8.19 to 10.86)

* Numbers in parenthesis indicate the maximum score of the outcome.

† Mean and SD in parenthesis.

**Table 5:** Parents’ outcomes at preintervention and postintervention and their differences (β coefficients) with 95% CIs estimated using linear regression models (n=478)

Outcome*	Pretest (T1)†	Post-test (T2)†	Estimated change (95% CI)
Positive and involved parenting (40)	18.6 (6.9)	29.2 (6.5)	10.6 (9.43 to 11.85)
Parental monitoring (40)	24.3 (5.0)	26.4 (7.8)	2.05 (0.86 to 3.23)
Total behaviour (50)	31.4 (5.7)	42.6 (4.2)	11.11 (10.20 to 12.02)
Discipline caregiver (80)	13.3 (12.5)	2.7 (2.8)	−10.60 (−12.23 to −8.96)
Depression (27)	8.1 (5.8)	2.9 (2.8)	−5.14 (−5.96 to −4.32)
Gender views and attitudes (32)	22.2	27.6	5.36 (4.55 to 6.16)
Financial protection	11.8 (4.8)	6.8 (6.2)	−4.97 (−5.98 to −3.97)

* Numbers in parenthesis indicate the maximum score of the outcome.

† Mean and SD in parenthesis.

Very similar means were found between adolescents and parents at baseline in all scales. After the intervention, a statistically significant positive difference in all scales was found. Among adolescents, greater improvements were found in positive and involved parenting (β=13.7; 95% CI 12.43 to 14.98), reduced harsh discipline (β=−8.69; 95% CI −10.45 to −6.94), improved youth behaviour (β=9.12; 95% CI 8.11 to 10.13) and resilience (β=9.53; 95% CI 8.19 to 10.86). In parents, the greater improvements were also in positive parenting (β=10.6; 95% CI 9.43 to 11.85), reduced harsh discipline (β=−10.60; 95% CI −12.23 to −8.96), improved youth behaviour (β=11.11; 95% CI 10.20 to 12.02) and improved financial coping (β=−4.97; 95% CI −5.98 to −3.97).

## Discussion

This study assessed the pilot implementation of the Parwarish (PLH-Teens) parenting intervention among disadvantaged families in informal urban settings—rural and remote tribal communities in India. We found positive improvements in all the psychometric measures among parents and young people with significant decreases in maltreatment reported by both youth and parents. The secondary outcomes of mental health, resilience, gender-equal attitudes and difficult behaviour also improved significantly among both parents and young people. These findings are important as the intervention was implemented in settings with typically high rates of child and youth maltreatment. Our findings align with the RCT and pre-post trials of PLH-Teens in other low-income settings, which show similarly positive outcomes using the same outcome measures.^13 18 19^

One of the factors most likely to have influenced the positive findings is that parents and young people were all highly disadvantaged and so started with high baselines (eg, depression or behaviour management), meaning that they had substantial capacity to benefit. Other contextual factors that may have strengthened the results were discussed in the process evaluation.^22^ These included the presence of implementing organisations that were known and trusted in the community, which likely enhanced recruitment and trust; implementation by lay community members, which may have increased relevance, micro-adaptations and acceptability of the group facilitation; and the responsiveness of implementers to community needs, such as offering a workshop on using the Right to Information Act when requested.

This intervention was facilitated by men and women (many of whom were married couples) who were predominantly peers (coresidents) of the communities who joined the Parwarish programme as participants. The participation of men as cofacilitators is likely to have strengthened the participation of fathers in the intervention groups. Other studies have similarly shown the importance of engaging with men when addressing violence in families in India.^5 38^ It also aligns with the findings of a recent systematic review of integrated domestic violence and reproductive health interventions in India, which found that all of the effective interventions used psychoeducation/education and skill building, both core components of the Parwarish intervention.^39^

Additionally, the Parwarish intervention is likely to have been effective through a combination of three key mechanisms. First, implementation using a group platform is likely to have triggered outcomes of itself, such as through providing opportunity for new peer friendships, mutual social support and new social networks. Further likely mechanisms explaining how psychosocial support groups improve social and mental health outcomes have been proposed in a scoping review by coauthors of this paper.^40^ Second, the participatory facilitation and role-plays may have supported participants to unearth knowledge within their group members, thus promoting self-belief, social capital and resilience skills.^14 41^ This participatory process has been shown to also build skills for non-violence, as shown in a recent narrative review of 42 programmes in LMICs, suggesting that participatory approaches can challenge prevailing norms and build new communication skills, which are key to changing attitudes and skills for non-violence.^42^ Third, the Parwarish curriculum includes content to support behavioural changes, such as increasing self-belief and identity, problem-solving, financial literacy and creating safe spaces.^14 24 43^ It also uses principles central to effective child and youth abuse prevention programmes, such as positive parenting and collaborative learning approaches that are also likely to have increased effectiveness.^14^

### Methodological considerations

This study has several strong design features. It is a ‘real-world’ trial adapted by local community members to low-income Indian contexts and implemented by local community members in the homes or courtyards of participating families, which assures that it can achieve meaningful outcomes outside of a trial setting. It was implemented with a modest budget (INR35 lakh or US$40 000), which facilitates its feasibility and provides assurance that it can be generalised and scaled in other low-income settings.^44^

There are several limitations to consider. First, the absence of a control group means that the observed changes cannot be interpreted as causal effects of the intervention. Second, research and implementation coordinators were employed by the same organisation (although working at different sites), which may have introduced bias. At the same time, the insider position of the evaluators also constituted a strength, as their familiarity with the local context and organisational processes facilitated pragmatic implementation, strengthened trust with implementation teams and allowed for iterative improvements throughout the intervention.

Third, the study was implemented across three diverse settings; however, the sample size was insufficient to permit site-specific analyses, limiting the ability to explore contextual differences. In addition, participants were clustered within sites, but clustering was not accounted for in the analysis due to the small number of clusters, which may have led to underestimation of SEs.

Fourth, no formal sample size calculation was conducted, as this was a pragmatic implementation study. Nevertheless, the relatively large sample size, the magnitude of the observed effects and the consistency of statistically significant findings across multiple validated outcomes provide some confidence in the observed patterns. However, as a pre-post study without baseline-adjusted analyses, the findings may also be affected by regression to the mean, whereby extreme baseline values move closer to the average over time independent of the intervention.

Finally, the study relied on self-reported outcomes, which is common in parenting intervention research^18 20^ but introduces the risk of measurement bias. Participants may have over-reported improvements due to social desirability or perceived expectations from facilitators, and the intervention may have influenced reporting practices rather than behaviour itself. To mitigate these risks, we triangulated findings by collecting reports from both adolescents and parents, conducted interviews in private settings and assured participants of confidentiality, and that their responses would not be shared with implementers during the study period.

## Conclusions

This study examined the pilot implementation and outcomes of the adapted PLH programme in three diverse communities in India. It provides preliminary evidence on feasibility and potential effectiveness, suggesting the Parwarish intervention may be effective at reducing harsh parenting and improving mental health and resilience for both young people and parents.

Our findings suggest that it is key for programmes that aim to reduce maltreatment of young people to embed initiatives within communities and institutions. This evaluation joins a suite of other studies that have evaluated the adaptations of PLH in diverse LMIC settings, including South Africa, China, Indonesia and the Philippines. All these studies have suggested this intervention can improve positive parenting and well-being for young people and parents in communities in South Asia. PLH-Teens merits further assessment and scaling in other settings.

## Supplementary material

10.1136/bmjopen-2025-113646 online supplemental file 1

## Data Availability

Data are available upon reasonable request.
