# Supplementary material for: Reducing violence for adolescents and their parents in three disadvantaged communities in North India: a pilot implementation study of the Parwarish (PLH-Teens) parenting programme
Source: BMJ Open. 2026 Jun 25;16(6):e113646. doi: 10.1136/bmjopen-2025-113646 (PMC13311703; doi:10.1136/bmjopen-2025-113646)
Supplement: online supplemental file 1 [file bmjopen-16-6-s001.docx]

**Supplementary Table 1**. Reliability checks of pre-post scales

|  | Cronbach’s alpha | Assessment |
| --- | --- | --- |
| **Parent or caregiver** | | |
| Child maltreatment^[[1]](#footnote-1)^ | 0.91 (0.89-0.93) | Excellent |
| Positive parental involvement^[[2]](#footnote-2)^ | 0.86 (0.83-0.88) | Very good |
| Positive parental supervision^2^ | 0.55 (0.46-0.62) | Less strong |
| Mental health^[[3]](#footnote-3)^ | 0.86 (0.83-0.88) | Very good |
| Financial coping^[[4]](#footnote-4)^ | 0.83 (0.79-0.86) | Very good |
| Gender equal attitudes^[[5]](#footnote-5)^ | 0.60 (0.52-0.67) | Satisfactory |
| **Young person** | | |
| Child maltreatment^1^ | 0.88 (0.86-0.90) | Very good |
| Young person behaviour^[[6]](#footnote-6)^ | 0.69 (0.63-0.75) | Acceptable |
| Positive parental involvement^2^ | 0.889 (0.87-0.91) | Very good |
| Positive parental supervision | 0.38 (0.26-0.49) | Poor |
| Young person behaviour5 | 0.71 (0.66-0.76) | Acceptable |
| Mental health^3^ | 0.81 (0.77-0.84) | Very good |
| Gender equal attitudes^5^ | 0.53 (0.43-0.61) | Less strong |
| Youth resilience^[[7]](#footnote-7)^ | 0.89 (0.86-0.91) | Very good |

1. ISPCAN Child Abuse screening tool (ICAST) [↑](#footnote-ref-1)
2. Alabama Parenting Questionnaire [↑](#footnote-ref-2)
3. PHQ9 Questionnaire [↑](#footnote-ref-3)
4. Family financial coping Questionnaire [↑](#footnote-ref-4)
5. Gender equal attitudes Measure [↑](#footnote-ref-5)
6. Strengths and Difficulties Questionnaire [↑](#footnote-ref-6)
7. Child Youth Resilience Measure [↑](#footnote-ref-7)
