# Supplementary material for: Reducing violence for adolescents and their parents in three disadvantaged communities in North India: a pilot implementation study of the Parwarish (PLH-Teens) parenting programme
Source: BMJ Open. 2026 Jun 25;16(6):e113646. doi: 10.1136/bmjopen-2025-113646 (PMC13311703; doi:10.1136/bmjopen-2025-113646)
Supplement: Supplementary file 2 [file bmjopen-2025-113646.reviewer_comments.pdf]

PEER REVIEW HISTORY

BMJ Open publishes all reviews undertaken for accepted manuscripts. Reviewers are asked to complete a checklist review form and are provided with free text boxes to elaborate on their assessment. These free text comments are reproduced below.

ARTICLE DETAILS

Title (Provisional)

Reducing violence for adolescents and their parents in three disadvantaged communities in North India - a pilot implementation study of the Parwarish (PLH-Teens) parenting program

Authors

Mathias, Kaaren R; Pillai, Pooja; Nayak, Prabhudutt; Singh, Pratibha; San Sebastian, Miguel

---

VERSION 1 - REVIEW

Reviewer 1
Name Reif, Lindsey
Affiliation Joan and Sanford I Weill Medical College of Cornell University, Global Health
Date 29-Dec-2025
COI None

Overall

This is a well written manuscript focused on assessing the impact of an evidence-based positive parenting program called Parwarish on reducing the experience of violence by parents toward children in families, and mental health, gender attitudes, financial coping, and resilience among both parents and children in several settings in Northern India. While this intervention has evidence shown to have an impact in other settings, this is the first study of implementing an adapted version of this intervention in India, which carries a high burden of violence against children and adolescents. It includes a thoughtful discussion on the implementation of this intervention in collaboration with the community to emphasize critical considerations for further scale-up in other settings.

Abstract

Minor edits:

-sentence structure for the first sentence of the introduction in the abstract needs editing. Re-state to emphasize that this is a critical public health issue ‘because’ India has the largest population of young people.

Introduction

Well written and summarizes the scope of the problem as well as previous studies of the original intervention.

Methods

Suggest adding a brief description of the training for the implementation team/coaches. This may have been included in the process evaluation paper, but a brief description is advised. Training for coaches/group leaders is an important aspect of the program’s success.

Minor edits:

Sentence structure edit to first sentence in participant recruitment paragraph.

Results

Minor edits:

-confirm correct +/- sign on ‘reduced harsh discipline’ experienced by young people matches text/table. Similarly, confirm the correct sign is reported in the results section of the abstract.

Discussion

Authors provide a thoughtful and thorough discussion to put the results in context. They include a concise and accurate summary of potential limitations and how they were addressed, and propose feasible considerations for expansion and scale up.

**Reviewer**	**2**
**Name**	**Chakraborty, Arup**
**Affiliation**	**Medical College and Hospital Kolkata, Community Medicine**
**Date**	**26-Feb-2026**
**COI**	**None**

Study type and design is not proper

Was there any sample size calculated?

Intervention detail needs to be mentioned (duration, method of education, frequency, monitoring etc.)

Key limitations of the study are lack of a comparison group. How do you generalize the finding of the study?

**Reviewer**	**3**
**Name**	**Marimirofa, Mercy**
**Affiliation and Research**	**Zimbabwe National Family Planning Council , Evaluation**
**Date**	**09-Apr-2026**
**COI**	**None**

The research is okay. However result presentation should clearly be done. The statistical component needs a relook although the analysis was proper to the type of data analysed. Which type of regression was used, the author did not mention

**Reviewer**	**4**
**Name**	**Dack, Kyle**
**Affiliation**	**University of Bristol, MRC Integrative Epidemiology Unit**
**Date**	**22-Apr-2026**
**COI**	**None**

Statistical review summary:

The reporting of the methods and results is clear and aligned with publishing guidelines. The interpretation is reasonable - pre-post designs have limits but the discussion is suitably cautious. However, the statistical analysis is slightly underspecified and most of my comments below relate to this.

Abstract:

1. "reduced harsh discipline ($\beta = 8.69$; 95% CI: -10.45, -6.94)" the coefficient is missing the negative sign.

Introduction:

2. The introduction provides a clear summary of the context, rationale, and aims of the study (no suggestions).

Methods:

3. Outcomes: the tools are described clearly, but are any of these more important than others, i.e. primary outcomes?

4. Trial registration is mentioned in the header of the manuscript, but not the main methods. It would be valuable to add it, and confirm whether there are any notable deviations, or none.

Statistical analysis:

5. The current model specification is somewhat unclear. I suggest fully describing all elements of the model, e.g. the IV, the DV, and what the coefficient represents. It is not clear from the current specification whether regression to the mean was handled (i.e. baseline adjustment), which is one of the major risks of bias in this study design.

6. -There is potential clustering at the three centres, was this explored, such as estimating ICC or comparing mean change per centre? If there is unaccounted clustering it could deflate the standard errors.

7. -The rationale provided for not adjusting for covariates could be expanded upon. In this design, confounding is not a dominant risk, as everyone received the intervention and as the authors state, the covariates are time-invariant. However, adjusting for them still has benefits, such as explaining variability and improving estimate precision.

Results:

8. " reduced harsh discipline (β = 8.69; 95% CI: -10.45, -6.94)" the coefficient is missing the negative sign.

Table 4/5.

9. -Please add the statistical method used to estimate the betas, e.g. linear regression, and the N. The table could be strengthened if mean statistics were presented alongside a measure of variance, such as standard deviation.

10. -I think the first column is reporting the maximum score in parentheses, which is very helpful, and suggest explaining this in a footnote or the table titles.

11. -The label "crude model" may make readers think this will be followed by an adjusted model. In the absence of this, I suggest just labelling it "estimated change" or "modelled change".

Discussion

12. There are limits to how pre-post designs can be interpreted causally, due to the lack of a comparator group, and the susceptibility to time-related biases (secular trends, regression to the mean). The discussion does not interpret the results too strongly, and the language used is suitably cautious. Because of this, the discussion seems suitable and I have no major suggestions. My only concerns regarding the validity relate to the previously mentioned queries regarding clustering and baseline adjustment.

VERSION 1 - AUTHOR RESPONSE

Reviewer: 1
Dr. Lindsey Reif, Joan and Sanford I Weill Medical College of Cornell University
Comments to the Author:

Overall
This is a well written manuscript focused on assessing the impact of an evidence-based positive parenting program called Parwarish on reducing the experience of violence by parents toward children in families, and mental health, gender attitudes, financial coping, and resilience among both parents and children in several settings in Northern India. While this intervention has evidence shown to have an impact in other settings, this is the first study of implementing an adapted version of this intervention in India, which carries a high burden of violence against children and adolescents. It includes a thoughtful discussion on the implementation of this intervention in collaboration with the community to emphasize critical considerations for further scale-up in other settings.

Abstract
Minor edits:
-sentence structure for the first sentence of the introduction in the abstract needs editing. Re-state to emphasize that this is a critical public health issue 'because' India has the largest population of young people.

--- Thank you. The abstract has been restructured to meet the journal's requirements, and that sentence has been removed.

Introduction
Well written and summarizes the scope of the problem as well as previous studies of the original intervention.

--- Thanks.

Methods
Suggest adding a brief description of the training for the implementation team/coaches. This may have been included in the process evaluation paper, but a brief description is advised. Training for coaches/group leaders is an important aspect of the program's success.
Minor edits:
Sentence structure edit to first sentence in participant recruitment paragraph.

--- Thanks. A brief description of the training has been added (page 12).

> *The study was implemented between June and October 2019. All the facilitators and coaches participated in 7 days training of the broad content which was delivered by the PLH-Teen group from South Africa which was run in Delhi. This was followed by three further days of training for the coaches. This training addressed both content of the modules (working through each of the modules during these days) as well as covering skills in interactive facilitation and group leadership skills. There was 100% attendance by facilitators and coaches (n=25) as well as implementers.*

--- Thanks. That sentence was unclear and has now been revised (page 10) to read:

> *Participants were recruited by facilitators through purposive sampling, by directly inviting known families with an adolescent living at home, and subsequent snowball*

sampling. Inclusion criteria were residence in the target areas; having at least one adolescent aged 12-18 years; having one or both parents acting as primary caregivers (residing in the same household for at least 4 nights a week); and willingness to participate in the two-hour weekly group sessions.

Results
Minor edits:
-confirm correct +/- sign on 'reduced harsh discipline' experienced by young people matches text/table. Similarly, confirm the correct sign is reported in the results section of the abstract.

--- Thanks. This has been revised and modified.

Discussion
Authors provide a thoughtful and thorough discussion to put the results in context. They include a concise and accurate summary of potential limitations and how they were addressed, and propose feasible considerations for expansion and scale up.

--- Thanks. The methodological considerations section has been revised to better reflect the study's potential limitations. We consider it challenging to propose specific recommendations for scale-up, as context is a key determinant of implementation. However, the intervention is designed to be adaptable to different settings. In the Conclusions section, we have added key considerations for implementers.

A key challenge for programmes that aim to reduce maltreatment of young people is how to embed initiatives such as Parwarish widely within communities and institutions, and thereby to scale them. It is also important to consider how to engage parents, particularly men, to sustain changes over the long term.

Reviewer: 2
Dr. Arup Chakraborty, Medical College and Hospital Kolkata
Comments to the Author:

Study type and design is not proper

--- We respectfully disagree with this comment. The study design is appropriate for the stated aim, which was to evaluate the *pilot implementation* of the Parwarish intervention in real-world settings. As described in the manuscript, we used a pre–post (before–after) study design without a control group, which is a recognized approach for feasibility and early effectiveness evaluations, particularly in implementation research and resource-constrained settings.

--- We explicitly justified this choice in the Methods section, noting that randomized controlled trials (RCTs) are often not feasible in early-stage, real-world program implementation due to logistical, ethical, and financial constraints. The intention of this study was not to establish causality but to generate preliminary evidence on potential effectiveness, feasibility, and scalability.

Was there any sample size calculated?

--- Thank you for raising this point. A formal sample size calculation was not conducted, as this study was designed as a pragmatic pilot implementation study rather than a hypothesis-testing trial. This is already presented in the Methods section.

--- Instead, the sample size was determined pragmatically based on implementation capacity and community engagement, with an initial target of approximately 250 families. Ultimately, 239 families participated.

--- We acknowledge that the absence of a formal sample size calculation is a limitation and has now been clarified in the Methodological consideration section. It reads now (page 22):

> *Fourth, no formal sample size calculation was conducted, as this was a pragmatic implementation study. Nevertheless, the relatively large sample size, the magnitude of the observed effects, and the consistency of statistically significant findings across multiple validated outcomes provide some confidence in the observed patterns.*

Intervention detail needs to be mentioned (duration, method of education, frequency, monitoring etc.)

---We have added further detail to address this suggestion. While the manuscript already details the intervention duration, methods, frequence in the Methods section (see Intervention page 12), we have added detail on the frequency of coaching and monitoring as provided below.

> *. The implementation team comprised of two programme coordinators (PS and PN) and three coaches who were also project leaders at each site. This group met fortnightly to discuss implementation, address any concerns and co-develop implementation processes. Coaches selected group facilitators with these inclusion criteria: effective communication skills, fluency in the local language, an equal mix of men and women (over half of group facilitators working together were married couples), having completed at least class 10 at high school and resident in the target community. A total of 19 group facilitators were identified and each led one or two groups. Coaches visited facilitators and their group sessions at least once a fortnight to conduct monitoring and fidelity assessments. In addition, they provided weekly support to facilitators, including discussions of implementation challenges and training on intervention content throughout the trial. Further details on implementation, fidelity, and processes are available in the published process evaluation paper (22).*

Key limitations of the study are lack of a comparison group. How do you generalize the finding of the study?

--- We agree that the absence of a comparison group limits causal inference and generalizability. This limitation is explicitly acknowledged in the manuscript, where we state that observed changes cannot be attributed solely to the intervention.

--- However, several points support the relevance and cautious generalizability of our findings:

1. Real-world implementation across diverse settings: The intervention was conducted in three distinct and disadvantaged contexts (urban informal settlements, rural agricultural areas, and tribal regions), increasing ecological validity.
2. Consistency with prior evidence: Our findings align with results from randomized controlled trials of the Parenting for Lifelong Health (PLH) programme conducted in other low- and middle-income settings.
3. Use of validated outcome measures: Standardized and previously validated psychometric tools were used, improving reliability and comparability.
4. Large and socioeconomically relevant sample: The study included highly disadvantaged populations, which are key target groups for such interventions.

--- We have been careful not to overstate generalizability and instead frame the findings as promising preliminary evidence. We explicitly recommend further “assessment and scaling in other settings”. to confirm effectiveness and support broader generalization (page 22).

Reviewer: 3
Dr. Mercy Marimirofa, Zimbabwe National Family Planning Council
Comments to the Author:

The research is okay. However result presentation should clearly be done. The statistical component needs a relook although the analysis was proper to the type of data analysed. Which type of regression was used, the author did not mention

--- Thank you for your comment. We appreciate your positive assessment of the overall research and statistical approach.

--- However, we find the comment regarding the presentation of results somewhat unclear. We would be grateful if you could specify which aspects of the results presentation require further clarification so that we can address them more precisely.

--- Regarding the statistical analysis, we would like to clarify that linear regression models were used to estimate changes between pre- and post-intervention outcomes, as described in the Methods section (page 16).

Reviewer: 4
Dr. Kyle Dack, University of Bristol
Comments to the Author:

Statistical review summary:
The reporting of the methods and results is clear and aligned with publishing guidelines. The interpretation is reasonable - pre-post designs have limits but the discussion is suitably cautious. However, the statistical analysis is slightly underspecified and most of my comments below relate to this.

Abstract:
1. "reduced harsh discipline (β = 8.69; 95% CI: -10.45, -6.94)" the coefficient is missing the negative sign.

--- Thanks for pointing this out. It has now been corrected.

Introduction:
2. The introduction provides a clear summary of the context, rationale, and aims of the study (no suggestions).

--- Thanks.

Methods:
3. Outcomes: the tools are described clearly, but are any of these more important than others, i.e. primary outcomes?

---Thanks for this suggestion, we included the primary outcomes in bold in Table 2 and have added text to clarify that these are the primary outcomes on page 14 to read:

> *The primary outcome measured was youth and parent reports of maltreatment (violence or abuse) i.e. IPSCAN and APQ.*

Trial registration is mentioned in the header of the manuscript, but not the main methods. It would be valuable to add it, and confirm whether there are any notable deviations, or none.

---Thank you for this suggestion. We have added text to confirm there were no notable deviation and also added the trial registration detail to the main methods (page 17).

> *Ethics approval for Protocol 191 was granted by the EHA Institutional Ethics Committee in March 2019. We completed prospective clinical trial registration with the Australasian clinical trials registry in June 2022 with the following identification: ACTRN12622000858796.*

Statistical analysis:
5. The current model specification is somewhat unclear. I suggest fully describing all elements of the model, e.g. the IV, the DV, and what the coefficient represents. It is not clear from the current specification whether regression to the mean was handled (i.e. baseline adjustment), which is one of the major risks of bias in this study design.

--- Thank you for this comment. We agree that clearer description of the model specification is important.

--- We have now revised the Methods section to explicitly describe the regression models used, including the dependent variables (post-intervention outcome scores), the independent variable (time: pre vs post), and the interpretation of the regression coefficient as the mean change in outcome between baseline and endline (page 16).

> *Linear regression models were used to estimate the mean change in outcome scores between pre- and post-intervention measurements, with time (baseline vs endline) as the independent variable and outcome scores as dependent variables; baseline values were not included as covariates since they were time invariant.*

--- Regarding regression to the mean, we acknowledge that this is a potential source of bias in pre–post study designs such as ours. In our analysis, we estimated change over time using

repeated measures without including baseline values as covariates (i.e., no baseline-adjusted model was applied). Therefore, regression to the mean cannot be fully ruled out. We have now clarified this point in the manuscript and explicitly acknowledged regression to the mean as a limitation of the study design (page 22).

> *However, as a pre–post study without baseline-adjusted analyses, the findings may also be affected by regression to the mean, whereby extreme baseline values move closer to the average over time independent of the intervention.*

6. -There is potential clustering at the three centres, was this explored, such as estimating ICC or comparing mean change per centre? If there is unaccounted clustering it could deflate the standard errors.

--- Thank you for this important observation. We acknowledge that participants were recruited from three different implementation sites, which introduces the potential for clustering effects.

--- However, given that the number of clusters was very small (n = 3), formal estimation of cluster-level effects such as intra-class correlation coefficients (ICC) or the use of multilevel modelling was not considered appropriate or statistically robust. Reliable estimation of ICCs generally requires a larger number of clusters.

--- We recognise that unaccounted clustering may have led to underestimation of standard errors and have now acknowledged this as a limitation in the revised manuscript (page 22).

> *Third, the study was implemented across three diverse settings; however, the sample size was insufficient to permit site-specific analyses, limiting the ability to explore contextual differences. In addition, participants were clustered within sites, but clustering was not accounted for in the analysis due to the small number of clusters, which may have led to underestimation of standard errors.*

7. -The rationale provided for not adjusting for covariates could be expanded upon. In this design, confounding is not a dominant risk, as everyone received the intervention and as the authors state, the covariates are time-invariant. However, adjusting for them still has benefits, such as explaining variability and improving estimate precision.

--- Thank you for this important point. We agree that, in some contexts, adjusting for covariates can improve precision by explaining part of the variability in the outcome.

--- However, this benefit depends on the strength of the association between the covariates and the outcomes. In our study, most available covariates were time-invariant and the sample was relatively homogeneous across key characteristics. Including such variables may have provided limited gain in precision and, in some cases, could introduce unnecessary model complexity, overfitting (especially with modest sample sizes) or reduce efficiency.

--- Given the pre–post design, where all participants received the intervention and the primary objective was to estimate overall change over time, we opted for a parsimonious modelling approach without covariate adjustment.

Results:

8. " reduced harsh discipline (β = 8.69; 95% CI: -10.45, -6.94)" the coefficient is missing the negative sign.

--- Thanks. Corrected.

Table 4/5.
9. -Please add the statistical method used to estimate the betas, e.g. linear regression, and the N. The table could be strengthened if mean statistics were presented alongside a measure of variance, such as standard deviation.

--- Thanks. Both suggestions have been added to the tables.

10. -I think the first column is reporting the maximum score in parentheses, which is very helpful, and suggest explaining this in a footnote or the table titles.

--- Thanks. Corrected.

11. -The label "crude model" may make readers think this will be followed by an adjusted model. In the absence of this, I suggest just labelling it "estimated change" or "modelled change".

--- Thanks. Corrected.

Discussion
12. There are limits to how pre-post designs can be interpreted causally, due to the lack of a comparator group, and the susceptibility to time-related biases (secular trends, regression to the mean). The discussion does not interpret the results too strongly, and the language used is suitably cautious. Because of this, the discussion seems suitable and I have no major suggestions. My only concerns regarding the validity relate to the previously mentioned queries regarding clustering and baseline adjustment.

---Thank you for your helpful comments.
